# Supplementary material for: HIVprotI: an integrated web based platform for prediction and design of HIV proteins inhibitors
Source: J Cheminform. 2018 Mar 9;10:12. doi: 10.1186/s13321-018-0266-y (PMC5845081; doi:10.1186/s13321-018-0266-y)
Supplement: Supplementary file 2 — Additional file 2. Source code of HIVProtI web server. [file 13321_2018_266_MOESM2_ESM.zip › HIVprotI_Source-code/hivprotI/weka-3-6-6/doc/weka/associations/tertius/Predicate.html]

Predicate


---


|  |  |  |
| --- | --- | --- |
| **Overview** \| **Package** \| **Class** \| **Tree** \| **Deprecated** \| **Index** \| **Help** \| **Weka's home** | |  |
| **PREV CLASS**   **NEXT CLASS** | **FRAMES**    **NO FRAMES**     **All Classes** |  |
| SUMMARY: NESTED \| FIELD \| CONSTR \| METHOD | DETAIL: FIELD \| CONSTR \| METHOD |  |


---


## weka.associations.tertius Class Predicate

```
java.lang.Object
  weka.associations.tertius.Predicate
```

**All Implemented Interfaces:**: java.io.Serializable, RevisionHandler

---

``` public class Predicate extends java.lang.Object implements java.io.Serializable, RevisionHandler ```

**Version:**
:   $Revision: 1.5 $

**Author:**
:   Peter A. Flach, Nicolas Lachiche

**See Also:**: Serialized Form

---

**Constructor Summary**

`Predicate(java.lang.String name,
int index,
boolean isClass)`


| **Method Summary** | |
| --- | --- |
| `void` | `addLiteral(Literal lit)` |
| `java.lang.String` | `description()` |
| `int` | `getIndex()` |
| `Literal` | `getLiteral(int index)` |
| `java.lang.String` | `getRevision()`             Returns the revision string. |
| `int` | `indexOf(Literal lit)` |
| `boolean` | `isClass()` |
| `int` | `numLiterals()` |
| `java.lang.String` | `toString()` |

**Methods inherited from class java.lang.Object**

`equals, getClass, hashCode, notify, notifyAll, wait, wait, wait`

**Constructor Detail**

### Predicate

```
public Predicate(java.lang.String name,
                 int index,
                 boolean isClass)
```


**Method Detail**

### addLiteral

```
public void addLiteral(Literal lit)
```

---


### getLiteral

```
public Literal getLiteral(int index)
```

---


### getIndex

```
public int getIndex()
```

---


### indexOf

```
public int indexOf(Literal lit)
```

---


### numLiterals

```
public int numLiterals()
```

---


### isClass

```
public boolean isClass()
```

---


### toString

```
public java.lang.String toString()
```

:   **Overrides:**: `toString` in class `java.lang.Object`

---


### description

```
public java.lang.String description()
```

---


### getRevision

```
public java.lang.String getRevision()
```

:   Returns the revision string.

    :   **Specified by:**: `getRevision` in interface `RevisionHandler`
    :   **Returns:**: the revision


---


|  |  |  |
| --- | --- | --- |
| **Overview** \| **Package** \| **Class** \| **Tree** \| **Deprecated** \| **Index** \| **Help** \| **Weka's home** | |  |
| **PREV CLASS**   **NEXT CLASS** | **FRAMES**    **NO FRAMES**     **All Classes** |  |
| SUMMARY: NESTED \| FIELD \| CONSTR \| METHOD | DETAIL: FIELD \| CONSTR \| METHOD |  |


---
